# Construction of high-density genetic map and QTL mapping of yield-related and two quality traits in soybean RILs population by RAD-sequencing

**DOI:** 10.1186/s12864-017-3854-8

**Published:** 2017-06-19

**Authors:** Nianxi Liu, Mu Li, Xiangbao Hu, Qibin Ma, Yinghui Mu, Zhiyuan Tan, Qiuju Xia, Gengyun Zhang, Hai Nian

**Affiliations:** 1 The State Key Laboratory for Conservation and Utilization of Subtropical Agro-Bioresources, South China Agricultural University, Guangzhou, 510642 People’s Republic of China; 2 The Key Laboratory of Plant Molecular Breeding, South China Agricultural University, Guangzhou, 510642 People’s Republic of China; 3 The Guangdong Subcenter of the National Center for Soybean Improvement, College of Agriculture, South China Agricultural University, Guangzhou, 510642 People’s Republic of China; 4 Joint Laboratory of Plant Breeding, South China Agricultural University-ShanDong Shofine Seed Technology Co. Ltd, Guangzhou, 510642 People’s Republic of China; 5 Beijing Genome Institute (BGI), Shenzhen, 518083 People’s Republic of China

**Keywords:** QTL, Yield-related traits, Quality traits, RAD-sequencing, Soybean

## Abstract

**Background:**

One of the overarching goals of soybean breeding is to develop lines that combine increased yield with improved quality characteristics. High-density-marker QTL mapping can serve as an effective strategy to identify novel genomic information to facilitate crop improvement. In this study, we genotyped a recombinant inbred line (RIL) population (Zhonghuang 24 × Huaxia 3) using a restriction-site associated DNA sequencing (RAD-seq) approach. A high-density soybean genetic map was constructed and used to identify several QTLs that were shown to influence six yield-related and two quality traits.

**Results:**

A total of 47,472 single-nucleotide polymorphisms (SNPs) were detected for the RILs that were integrated into 2639 recombination bin units, with an average distance of 1.00 cM between adjacent markers. Forty seven QTLs for yield-related traits and 13 QTLs for grain quality traits were found to be distributed on 16 chromosomes in the 2 year studies. Among them, 18 QTLs were stable, and were identified in both analyses. Twenty six QTLs were identified for the first time, with a single QTL (*qNN19a*) in a 56 kb region explaining 32.56% of phenotypic variation, and an additional 10 of these were novel, stable QTLs. Moreover, 8 QTL hotpots on four different chromosomes were identified for the correlated traits.

**Conclusions:**

With RAD-sequencing, some novel QTLs and important QTL clusters for both yield-related and quality traits were identified based on a new, high-density bin linkage map. Three predicted genes were selected as candidates that likely have a direct or indirect influence on both yield and quality in soybean. Our findings will be helpful for understanding common genetic control mechanisms of co-localized traits and to select cultivars for further analysis to predictably modulate soybean yield and quality simultaneously.

**Electronic supplementary material:**

The online version of this article (doi:10.1186/s12864-017-3854-8) contains supplementary material, which is available to authorized users.

## Background

Soybeans [*Glycine max (L.) Merrill*] contain complete protein and oil, providing all the essential amino acids necessary for the human diet [1]. Hence, a great effort has been made to increase soybean yield, while maintaining a high level of quality characteristics [2]. Yield and quality-related traits of soybean are quantitative traits that are controlled by a combination of genetic and environmental factors [3].

The genetic maps with traditional molecular markers including restriction fragment length polymorphism (RFLP), simple sequence repeats (SSR) and amplified fragment length polymorphism (AFLP) have been traditionally used to identify the genetic basis of complex traits in plants [4–7]. However, conventional molecular markers often display a low density and are unevenly distributed throughout the whole genome. Therefore, the genetic maps developed using these molecular markers have limited both the efficiency and accuracy of QTL positioning. Recently, with the rapid development of high-throughput sequencing technology, single nucleotide polymorphism (SNP) markers have emerged as new molecular markers of choice because of their high-density and relatively even distribution across plant genomes. Further, they have resolved many of the problems associated with the efficiency and accuracy of QTL mapping [8–12]. Several new technologies for SNP genotyping have been developed over the last few years. A high-throughput method for genotyping recombinant populations utilizing whole-genome resequencing to construct a dense genetic map using recombination bins as markers was developed by Huang et al. [13]. Restriction-site associated DNA sequencing (RAD-seq), was one of the next generation sequencing (NGS) methods, has been effectively applied in high-throughput SNP marker discovery and quantitative trait loci (QTL) analysis including the mapping of quality and agronomic trait loci in soybean [14].

Based on these new technologies for SNP genotyping, numerous QTLs associated with yield or quality traits have been identified in soybean [15–17]. For example, Kim et al. evaluated two populations for seed yield and other agronomic traits using 1536 SNP markers. In total, 8 QTLs for plant height and 3 QTLs for seed yield were identified [18]. In another study, two QTLs for protein content and six oil content QTLs were identified by Akond and colleagues using a RIL population derived from a cross of PI43848913 × Hamilton [19]. Further, a high density map was developed using the 5376 SNP markers from the Illumina Infinium BeadChip array. In addition, one protein and 11 oil content QTLs were detected in the MD96–5722 by ‘Spencer’ RILs population [20]. Hwang et al. detected 40 SNPs associated with seed protein content and 25 SNPs associated with seed oil content. Among these markers, 7 SNPs were found to be significantly associated with both protein and oil content [21].

The objectives of this research reported here were (1) to develop a high-density soybean molecular genetic bin map with the RAD-seq method, and (2) to map QTLs for yield and quality-related traits in the RIL population and compare these data with previous research (http://www.soybase.org), (3) to determine if any QTLs were identified in both years and were co-localized with any other trait-related QTLs, (4) to select candidate genes that may influence both yield and quality using Gene Ontology (GO) enrichment analysis.

## Methods

### Plant materials and field trials

A RIL population was developed from a cross between Zhonghuang 24 (female parent) and Huaxia 3 (male parent) using a modified single seed method [22]. Zhonghuang 24 is a variety with high-oil content adaptive to Huang-Huai-Hai region. Huaxia 3 was derived from a cross between ‘Guizao 1’ and ‘BRSMG68 (Brazilian variety)’ that is a high-yielding soybean cultivar. The 164 F_8_ RILs were grown together with both parents at the Zengcheng Experimental Station (South China gricultural University, Guangzhou, China) following a randomized complete block planting with three replications in the summer of 2012. Each plot contained 10 plants per row, with 0.5 m between rows and 0.1 m between plants. The 146 F_11_ RILs were grown using the same methods in the same location in 2015. Field management followed normal soybean production practices for the area.

### Measurement of yield-related and two quality traits

The five plants in the middle of each row were individually harvested to score the following traits: plant height (PH), number of nodes (NN), number of branches (BN), number of effective pods (EP), number of invalid pods (IP), 100-seed weight (SW), seed protein content (Pro) and seed oil content (Oil). PH was measured in mature plants as the distance (cm) from the cotyledonary node to the top node of the main stem. NN was measured by counting the number of nodes from the cotyledonary node to the top of the main stem. BN was determined by counting the number of branches with podding on the main stem. EP were obtained by counting the number of pods with more than one filled seed per pod. IP were obtained by counting number of pods that did not contain seed. SW was measured by weighing 100 random filled seeds. 50 g of seed from each line were used for protein and oil determination by an Infratec 1241 Grain Analyzer based on 10% moisture.

Frequency distribution and correlation analysis for the parental and RIL population were analyzed with the SPSS statistics 17.0 and Microsoft Excel 2007.

### Genetic map and QTL detection

#### SNP genotyping

All the genotyping work was conducted at the Beijing Genome Institute (BGI) Tech, Shenzhen, China. The soybean reference genome from Williams 82 was used for read mapping with SOAP software [23]. Input data for SNP calling with realSFS was prepared by SAMtools [24]. According to site frequency at every site, population SNP calling was performed with realSFS. The likelihoods of genotypes for each individual were integrated and extracted as candidate SNPs and then filtering these SNPs using the following criteria: 40 ≤ depth ≤ 2500,sites with a probability ≥ 95%. The homozygous genotype of parents and their populations were obtained based on the high fidelity-SNPs. According to the sliding window approach, we chose to include 15 SNPs per window, identifying the genotype for each window and the exchange sites for each individual when sliding a SNP every time, and then using the genotype for each individual to generate bin information [13].

#### QTL analysis

A high-density genetic map was constructed using MST software (http://alumni.cs.ucr.edu/~yonghui/mstmap.html). The composite interval mapping (CIM) method was employed to scan QTLs. The LOD thresholds for QTL significance were determined by a test (1000 replications) with a genome-wide at the 5% level of significance to judge whether there exist QTL. The location of a QTL was described according to its LOD peak location and the surrounding region with 95% confidence interval calculated using WinQTLCart software [25]. Running result of software can show additive effects of QTLs and phenotypic variation. The LOD values were shown in Additional file 1. QTL mapping results were comprehensively compared to Soybase (http://www.soybase.org/).

#### Method for naming QTLs

All QTLs were named according to Cui et al. as follows [26]: initial ‘q’ denotes ‘QTL’; the letters following it are the abbreviation of the corresponding traits; the next number is the soybean chromosomes on which the corresponding QTL is distributed; then, ‘a’ and ‘b’ represent whether the QTL was identified in 2012 and 2015, respectively; if more than one QTL for a certain trait was dispersed along a certain chromosome, a serial number, viz.-1, 2, etc., is used after ‘a’ or ‘b’ to describe their order.

## Results

### Phenotypic analysis of the RIL population

**Fig. 1:**
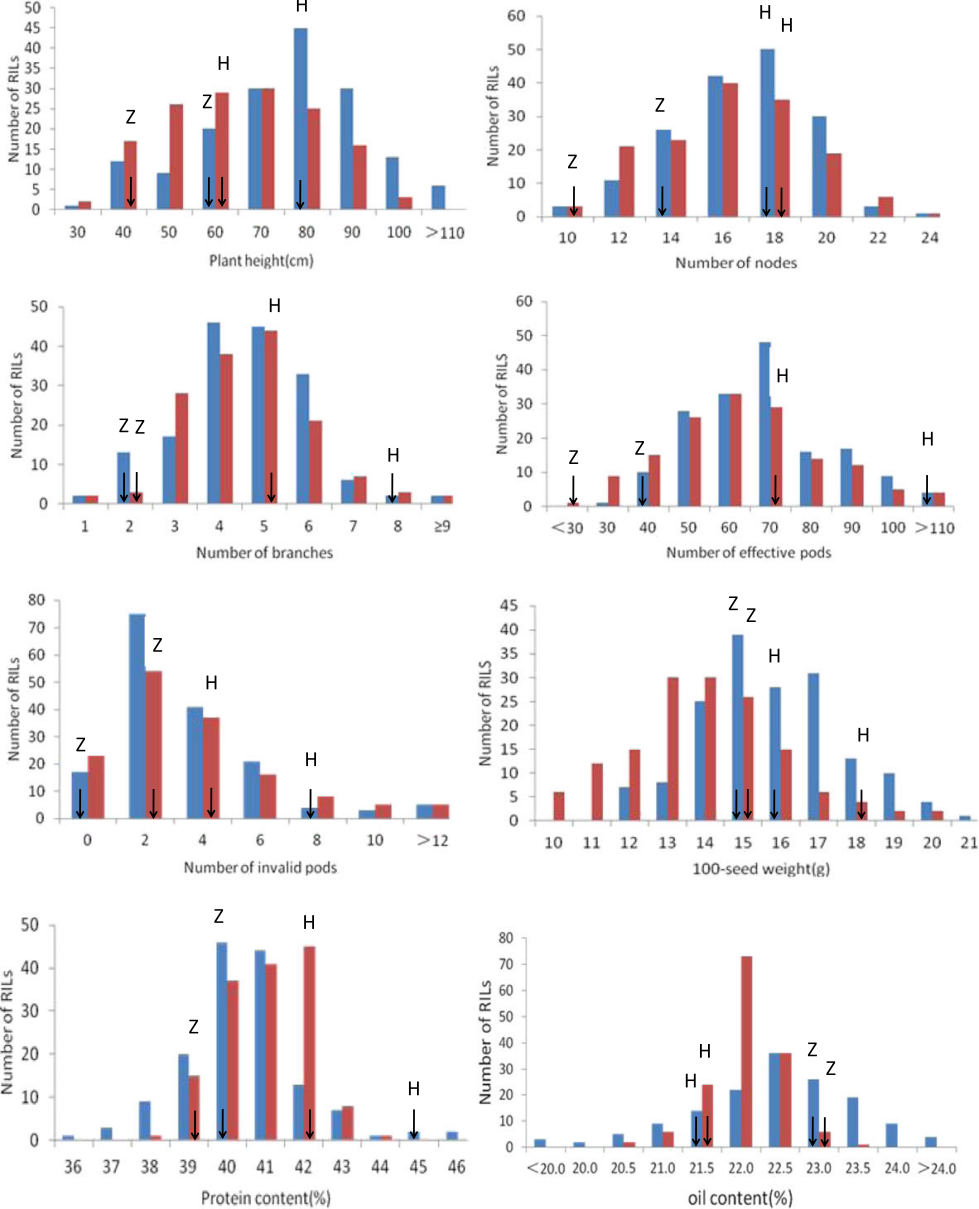
Frequency distribution for eight traits in 2012 (in *blue*) and 2015 (in *red*)

Most of the traits of Huaxia 3 showed higher values compared with those of Zhonghuang 24, providing ideal material for population construction and QTL analysis, with the exception of oil (Additional file 2). Figure 1 shows the frequency distribution for eight traits in 2 years. Phenotypic values were found to be continuous with normal or skew normal distributions. Transgressive segregation in the RILs was shown for eight traits, suggesting that alleles with positive effects on the measured traits are distributed among the parents.

The correlation analysis showed that most of the yield-related traits were correlated with each other in both years (Table 1). PH was positively correlated with NN, BN, EP, IP and SW, except for EP and IP in 2012 and SW in 2015 where it was not significant. NN also showed significant positive correlations with BN and EP in both years, but no correlation was detected with SW. Significant negative correlations were found for SW with BN and EP, ranging from *r* = −0.215^**^ to *r* = −0.327^**^in both years, but have a significant positive correlation with protein (*r* = 0.245^**^) in 2012. Most previous studies reported that there is a strong negative correlation between seed protein and seed oil content [20, 27]. In our study, a highly significant negative correlation (*r* = −0.775^**^, *r* = −0.761^**^) was observed between protein and oil in both years.

**Table 1:** Phenotypic correlations between yield-related traits and quality traits in 2012 and 2015

Traits	PH(2012^a^/2015^b^)	NN(2012^a^/2015^b^)	BN(2012^a^/2015^b^)	EP(2012^a^/2015^b^)	IP(2012^a^/2015^b^)	SW(2012^a^/2015^b^)	Pro(2012^a^/2015^b^)	Oil(2012^a^/2015^b^)
PH(2012^a^/2015^b^)	1							
NN(2012^a^/2015^b^)	0.788^**^/0.750^**^	1						
BN(2012^a^/2015^b^)	0.337^**^/0.249^**^	0.365^**^/0.313^**^	1					
EP(2012^a^/2015^b^)	0.113/0.339^**^	0.237^**^/0.495^**^	0.653^**^/0.514^**^	1				
IP(2012^a^/2015^b^)	−0.037/0.250^**^	−0.011/0.323^**^	0.337^**^/0.392^**^	0.455^**^/0.417^**^	1			
SW(2012^a^/2015^b^)	0.233^**^/0.134	0.062/0.004	−0.215^**^/−0.267^**^	−0.327^**^/−0.297^**^	−0.177^*^/−0.132	1		
Pro(2012^a^/2015^b^)	−0.054/0.094	−0.048/0.011	0.057/−0.040	−0.025/0.008	0.152/−0.057	0.245^**^/0.153	1	
Oil(2012^a^/2015^b^)	0.139/−0.016	0.153/−0.022	−0.017/0.021	0.065/−0.051	−0.186^*^/0.050	−0.102/0.040	−0.775^**^/−0.761^**^	1

*Abbreviations*: *PH* plant height, *NN* number of nodes, *BN* number of branches, *EP* number of effective pods, *IP* number of invalid pods, *SW* 100-seed weight, *Pro* seed protein content, *Oil* seed oil content

**Means significant correlation at *P* < 0.01;* Means significant correlation at *P* < 0.05

^a^The data used was generated in summer 2012

^b^The data used was generated in summer 2015

### High-density SNP linkage map construction

Based on 0.2× RAD-seq (restriction-site associated DNA sequencing) of the Zhonghuang 24 and Huaxia 3 RIL population, 57.40G sequence reads were obtained and the average read number was 311.97 M. Half of them have more than 200 M reads. According to this data, a total of 47,472 high-quality polymorphic SNP sites were detected for the RILs. All of the SNP sites in the RILs were integrated into a recombination bin unit, and 2639 recombinant bins were obtained. The average physical length of the bins was 360.01 kb, ranging from 20.01 kb to 17.43 Mb. A total of 1126 bins’ length were less than 100 kb, 609 bins ranging from 100 kb to 200 kb, 291 bins from 200 kb to 300 kb, 175 bins from 300 kb to 400 kb and 438 bins above 400 kb. Based on the genotypes of 2639 bins, a high-density bin linkage map was constructed covering 2638.24 cM, with an average distance of 1.00 cM between adjacent markers. For each chromosome, the average genetic distance between adjacent bins ranged from 0.67 to 1.51 cM (Table 2). Therefore, the linkage map constructed with recombination bins resulted in well-distributed linkage distances and has higher resolution than conventional maps.

**Table 2:** Description of characteristics of 20 chromosomes in the high-density genetic map

Chr^a^.	SNP number	Bin number	Linkage distance (cM)	Distance between adjacent bins (cM)
1	2384	116	128.799	1.11
2	1671	134	121.734	0.91
3	2344	144	134.913	0.94
4	1622	112	108.903	0.97
5	1117	102	117.605	1.15
6	3846	142	158.509	1.12
7	2219	137	121.151	0.88
8	1152	145	185.748	1.28
9	3416	176	151.747	0.86
10	1399	128	133.114	1.04
11	1689	124	112.662	0.91
12	648	85	128.605	1.51
13	1776	161	162.947	1.01
14	3496	116	132.173	1.14
15	4554	138	133.899	0.97
16	3943	110	118.263	1.08
17	2148	125	115.873	0.93
18	4421	186	124.441	0.67
19	934	120	114.751	0.96
20	2693	138	132.404	0.96
Total	47,472	2639	2638.241	1.00

^a^Chr indicates chromosome

### QTL analysis for yield-related traits

**Fig. 2:**
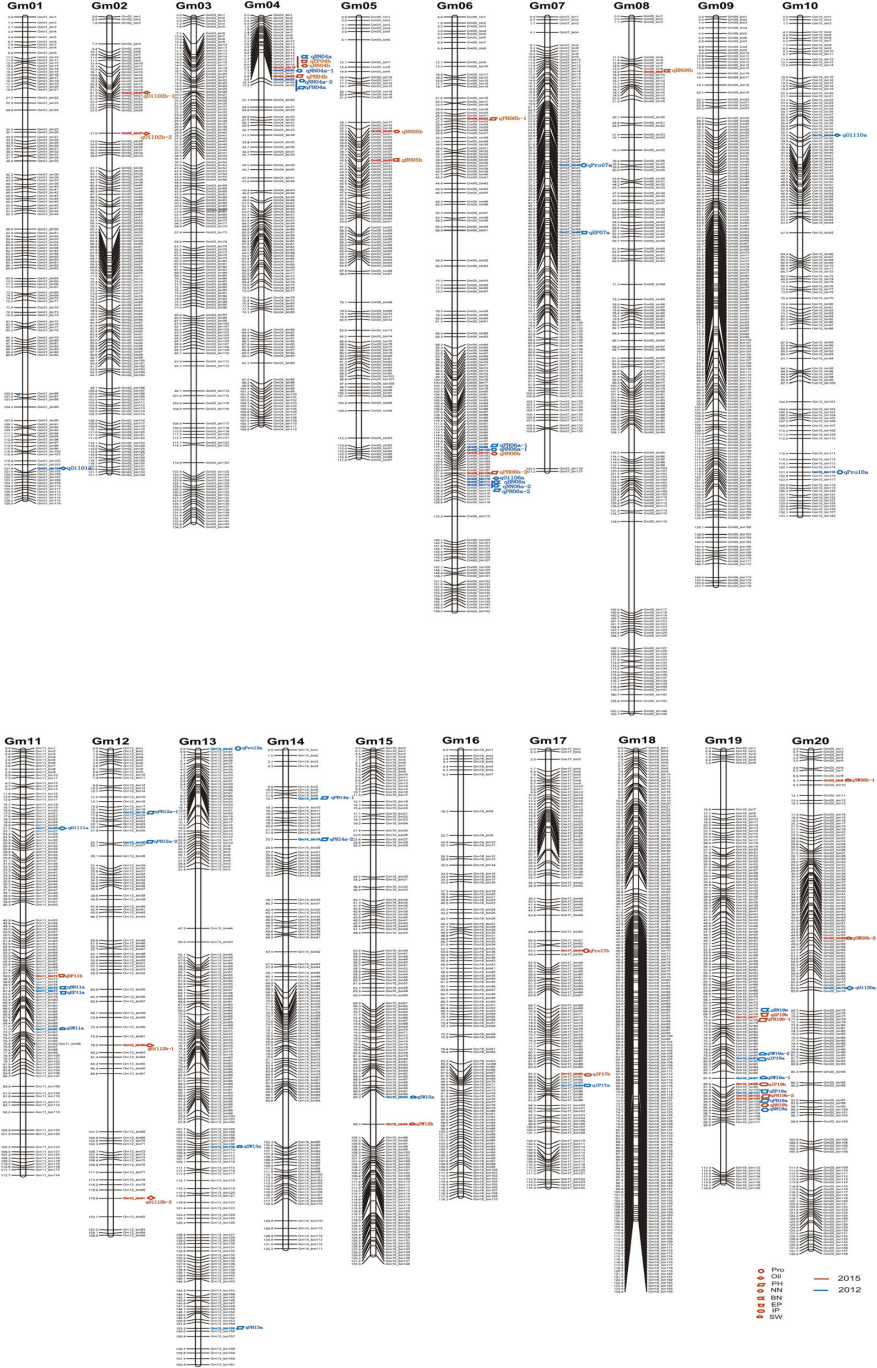
The positions of QTLs for eight traits. 60 QTLs for eight traits identified across 2 years are depicted in different shapes on the right side of each linkage group. 36 QTLs identified in 2012 are *colored in blue* and 24 QTL identified in 2015 are *highlighted in red*

Forty-seven QTLs associated with yield-related traits including PH, NN, BN, EP, IP and SW, were identified on 13 chromosomes (Chr04, Chr05, Chr06, Chr07, Chr08, Chr11, Chr12, Chr13, Chr14, Chr15, Chr17, Chr19, Chr20) (Fig. 2). A single QTL explained 3.78% (*qPH13a*)-32.56% (*qNN19a*) of phenotypic variance. Among the QTLs, 28 were identified on ten chromosomes in 2012. The most prominent QTL with the highest LOD score (15.63) was identified in a 56 kb region, which we designated *qNN19a,* explained 32.56% of phenotypic variation and displayed a negative additive effect, mainly with the positive allele from the male parent Huaxia 3. Nineteen QTLs on nine chromosomes were detected in 2015, and *qPH19b-2* has the most significant LOD score (10.34), explaining 24.49% of phenotypic variation and showed negative additive alleles from the male parent Huaxia 3. Of these QTLs, 24 were in agreement with earlier reports and 23 QTLs were found to be novel (Additional file 3 and Table 3). Eight QTL clusters responsible for more than two traits were detected on four different chromosomes (Additional file 4). A total of 18 QTLs were stable across both years. Thirty-four of these QTLs had a positive additive effect, which were contributed from the female parent Zhonghuang 24, whereas 13 QTLs had a negative effect, with additive alleles from the male parent Huaxia 3.

**Table 3:** Novel QTLs detected in Zhonghuang 24 × Huaxia 3 RILs population in 2 years

QTL Name	Chr^a^: Physical position	LOD^b^	Additive effect	R^2^ (%)^c^
qPH04a^d^	Chr04:3,836,380–5,131,478	8.21	7.73	15.71
qPH12a-1	Chr12:3,159,855–3,277,139	2.63	3.83	4.30
qPH12a-2	Chr12:4,498,030–4,639,202	3.97	4.61	6.53
qPH14a-2	Chr14:4,294,621–4,447,181	2.85	3.90	4.59
qPH04b^d^	Chr04:3,815,206–3,836,379	9.56	8.78	21.53
qPH06b-1	Chr06:4,124,778–4,212,333	2.70	−4.03	5.55
qNN04a-1^d^	Chr04:3,740,934–3,781,822	5.29	0.83	9.10
qNN04a-2	Chr04:3,836,380–5,131,478	5.09	0.86	9.59
qNN19a^d^	Chr19:45,081,886–45,138,371	15.63	−1.48	32.56
qNN04b^d^	Chr04:3,657,048–3,740,933	7.78	1.33	17.68
qNN19b^d^	Chr19:45,081,886–45,138,371	3.15	−0.76	6.60
qNN05b	Chr05:3,507,805–3,602,613	3.05	0.75	6.41
qBN04a	Chr04:3,657,048–3,740,933	6.64	0.55	13.44
qBN11a	Ch11:15,949,203–16,152,966	2.62	0.32	4.97
qBN05b	Chr05:5,079,314–5,122,378	3.52	0.46	9.74
qBN08b	Chr08:2,480,340–2,712,994	2.61	−0.37	6.29
qEP07a	Chr07:15,331,902–15,518,862	2.79	−5.35	6.78
qEP11a^d^	Chr11:16,152,967–16,449,587	3.07	5.52	6.96
qEP04b	Chr04:3,657,048–3,740,933	2.56	5.18	6.17
qEP11b^d^	Chr11:15,328,134–15,527,096	3.78	6.05	9.31
qSW20b-1	Chr20: 811,292–1,045,131	2.92	0.60	7.81
qIP17a^d^	Chr17:38,624,564–38,676,793	2.70	0.8285	5.80
qIP17b^d^	Chr17:38,167,697–38,257,004	2.78	0.938	6.89
qPro10a	Chr10:47,767,595–47,857,932	4.06	−0.47	9.49
qPro13a	Chr13: 19,893,079–20,804,523	3.39	0.42	7.91
qPro17b	Chr17: 11,691,747–11,743,779	3.43	−0.34	9.29

^a^Chr indicates chromosome

^b^LOD indicates the logarithm of odds score

^c^Percentage of phenotypic variation explained

^d^marked by QTL name indicates a new, stable QTL that was detected in both years

### QTL analysis for quality traits

A total of 13 QTLs were associated with quality traits on ten different chromosomes (Chr01, Chr02, Chr06, Chr07, Chr10, Chr11, Chr12, Chr13, Chr17, Chr20) in both growing seasons (Fig. 2). Three QTLs for protein content were identified on Chr07, Chr10 and Chr13 in 2012, respectively. Five QTLs for oil content were identified on Chr01, Chr06, Chr10, Chr11 and Chr20, with the phenotypic variance effect ranging from 6.76% (*qOil11a*) to 13.30% (*qOil01a*). Four QTLs (*qPro07a, qPro13a, qOil06a, qOil20a*) showed positive additive effects ranging from 0.27 (*qOil20a*) to 0.42 (*qPro13a*), while the other four QTLs (*qPro10a, qOil01a, qOil10a, qOil11a*) showed negative additive effects that were from −0.27 to −0.47. A QTL (*qPro17b*) for protein content was detected in a 52 kb region on Chr17, explaining 9.29% of phenotypic variation in 2015. In addition, four QTLs on Chr02 and Chr12 were identified for oil content, which individually explained 7.52% (*qOil02b-1*) and 12.49% (*qOil02b-2*) of the phenotypic variation. Within these QTLs, three of them had positive additive effects, indicating that the female parent, Zhonghuang 24, contributed the trait for increased oil content. A total of ten QTLs were reported in prior studies, and three new QTLs were identified for the first time in the present study.

### The Gene ontology enrichment analysis base on QTL hotpot

It was noteworthy that an important QTL hotspot was mapped in a physical position between 43,923,975 and 45,138,371 bp on Chr19. Seven QTLs associated with five traits that explained up to 32.56% of phenotypic variation, were all detected within this genomic region that was previously reported to be associate with seed weight, protein and oil in several different studies. In order to gain an in- depth understanding of which genes/QTLs were related to yield and quality in this region, we retrieved gene calls and annotations using Glyma.Wm82.a1.v1.1 gene model from SoyBase (https://soybase.org/SequenceIntro.php#mapscompare). A total of 139 genes were found within this region using Gene Ontology enrichment analysis, and among them, 51 annotated genes were closely related to yield or quality, which could be classified into five groups (Additional file 5). The first group contains 13 genes associated with phytohormone regulation, including hormones such as auxin, abscisic acid and ethylene, which play an essential role in coordination of in vitro and in vivo regulation mechanisms to simultaneously improve yield and quality [28]. The second group is comprised of 19 genes that are associated with metabolic processes, including carbohydrate metabolism, lipid metabolism, fatty acid catabolism and brassinosteroid metabolism, which are known to have an effect on the growth and development of soybean. The third group contains 6 genes associated with protein phosphorylation, which could be related to functional properties of food protein. Next, the fourth group is made up of 16 genes that are associated with cellular processes, including cell differentiation, cell proliferation, multicellular organism reproduction, and cell growth, which may have positive consequences for grain yield and quality in plants [29]. The fifth group consists of 16 genes associated with organ morphogenesis, including the development of root, stamen, leaf and seedling, etc., even directly influence on soybean yield and quality.

## Discussion

### Main effect factors for QTL mapping

The utility of QTL mapping is to obtain valuable alleles and understand genetic mechanisms, thus promoting genetic improvement of soybean by molecular methods, which is one of the main objectives in soybean breeding. Parental genetic diversity, environmental effects, and marker density are the main factors affecting QTL mapping [30]. In this study, the parents of the RIL population are derived from geographically distinct locations. Zhonghuang 24 is a main variety grown in central China, while male parent, Huaxia 3, is derived from Brazilian soybean germplasm that have high yield and become the main variety grown in southern China. Our data indicated that there were more differences in yield and quality-related traits between Zhonghuang 24 and Huaxia 3, relative to other similarly performed studies. Thus, the detected QTLs of these traits could be more useful for soybean improvement. In addition, quantitative traits can be strongly affected by environment factors [31]. In order to find QTLs that are stably expressed across environments, we chose two non-consecutive years including 2012 that was determined to be a suitable climate and 2015, which experienced greater than rainfall throughout all growth stages. According to Guangzhou Meteorological Service (http://www.gz121.gov.cn/), the total rainfall from July to October was 433 mm in 2012, while 1023 mm for the same period in 2015. Under these conditions, the QTLs identified in both years can be considered robust and environmentally stable. Furthermore, QTL mapping based on the resequencing genotyping method resulted in the integration of a total of 47,472 SNPs into 2639 recombination bin units. This was used to construct a high-density bin linkage map with an average distance of 1.00 cM between adjacent markers. The map has well-distributed linkage distances and higher resolution than the conventional map, making QTL mapping more accurate and reliable.

### Comparison of the present study with previous research

In the present study, 14 QTLs were identified for PH, explaining 3.78 to 28.01% of phenotypic variation across the two growth seasons, of which, *qPH19a* was major QTL associated with PH and was detected in both years. This QTL has been previously reported by Lee et al. and Specht et al. [32, 33]. It is worth noting the importance of the novel QTLs (*qPH04a, qPH04b*) on Chr04 identified in this study, because they expressed across both years and accounted for 15.71 and 21.53% of phenotypic variation, respectively. Three QTLs (*qPH06a-1, qPH06a-2, qPH06b-2*) were identified on Chr06, which were in similar regions of those previously reported by Wang et al. and Gai et al. [34, 35], respectively. Four novel QTLs (*qPH06b-1, qPH12a-1, qPH12a-2, qPH14a-2*) were identified on Chr06, Chr12 and Chr14 for PH. NN was found to be influenced by nine distinct QTLs distributed across four chromosomes. The QTL detected on Chr04 in 2012, with an interval of 3,740,934–3,781,822 bp, was in a similar region (3657048–3,740,933 bp) to another one identified in 2015, and it is likely that they are the same. Two QTLs were identified on Chr19, *qNN19a* and *qNN19b*, which were consistent in both years and explained up to 32.56% of phenotypic variation. Interestingly, no similar positions were found for NN in prior studies. BN, a key constituent of soybean yield, has been studied extensively. Some researchers think that increasing production could be achieved through adjusting the branching number, and was confirmed by Panthee et al. [36]. In their study, sd yld24–1 was mapped for yield traits with satt076 on Chr19. Interestingly, *qBN19a* which controls the number of branches in our study falls within this interval. Moreover, sd wt4–1 and sd wt11–1 for seed weight were identified by Maughan and Lee [37, 38], which was located at the same position as *qBN11a, qEP11a,* and *qEP11b* on Chr11 in this study. Three other novel QTLs (*qBN04a, qBN05b, qBN08b*) for BN were detected on Chr04, Chr05, and Chr08, accounting for 6.29 to 13.44% of phenotypic variation. Pod number and 100-seed weight are important parameters in measuring soybean yield and controlled by multiple genes. Two QTLs, *qEP19a* and *qIP19b,* on Chr19 were found to be associated with pod number during both years, and were located in the same region as those previously reported by Zhang et al. [39]. Moreover, *qSW19a-1* was shown to be associated with 100-seed weight, and is also mapped on Chr19 near this interval. Orf et al. reported a fine-mapped, 100-seed weight QTL located on Chr15, which just overlapped the intervals of the QTL for SW detected in both years in the present study [40].

In our study, a total of 4 protein content QTLs and 9 seed oil content QTLs were identified in 2 years. Three QTLs (*qPro10a, qPro13a, qPro17b*) were found to be novel, and no similar position has been identified previously for protein content. Ten of the 13 QTLs relevant to protein or oil content detected in the present study were consistent with previous research, and some of them shortened the interval. For example, A QTL associated with oil content, *qOil06a,* was found on Chr06 (37764770–38,299,977 bp). Palomeque et al. also reported that a QTL for oil content fell within the same interval, and a similar locus regarding seed oil and ‘oil plus protein’ related traits was also published by other researchers [41, 42], which indicated that this QTL is stable and may have pleiotropic effects. Meanwhile, three other QTLs (*qPH06a-2, qNN06a-2, qBN06a*) for yield-related traits were mapped to a similar region identified in our study, which explained 6.65 to 19.77% of phenotypic variation, respectively. *qOil20a* was mapped in a 39 kb region to bin 73 on Chr20 (34770628–34,809,740 bp), which falls within the same region identified by both Qi et al. and Reinprecht et al. [43, 44]. Moreover, *qSW20b-2* (33207531–33,259,106 bp) for yield was also located near this position, suggesting that these two aforementioned regions should be of great value for genetic improvement of both soybean yield and quality. The remaining QTLs associated with protein or oil content in agreement with those of previous studies are presented in Additional file 3 [27, 45–50]. The coincidence of QTL across different genetic backgrounds not only reveals the stability and reliability of the QTL detected herein but also highlights the significance of these regions in marker breeding works designed to develop higher protein or oil soybean cultivars.

### Important QTL hotspots

Most of the QTLs were clustered in eight genomic regions, particularly on Chr04, Chr06, Chr11 and Chr19 (Additional file 4). These QTLs hotspots included at least two traits such as PH, NN and SW, and was previously reported to be associated with some other traits in different genetic sources. Four QTLs for yield-related traits were mapped in two intervals of 3,657,048–3,781,822 bp on Chr04, which explained 6.17–17.68% of phenotypic variation. These QTLs have not been published and add to the growing knowledge on the genetic control of these traits. Three other QTLs were also detected on Chr04 (3815206–5,131,478 bp) explaining the range of phenotypic variation (9.59–21.53%). However, this region was reported to be associated with seed protein and seed weight in some earlier studies [33, 40]. Seven QTLs for PH, NN, BN, and oil were identified in two regions (18376759–19,504,937 bp, 37,764,770–41,420,709 bp) that were separated by a distance of more than 7 cM on Chr06, and accounted for 5.18–19.77% of phenotypic variation. Previously, Sun et al. located two QTLs for pod number on Chr06 near these two regions [51]. The first region on Chr06 in the present study has been shown to be associated with different traits by other researches [42, 45, 52]. Moreover, Chen et al. found that two QTLs for pod number and seed oil plus protein were consistent with the second region on Chr06 in our study [42]. More seed weight, protein and oil content QTLs were mapped to this locus in previous studies [17, 41, 45, 53, 54]. Three QTLs for BN and EP were identified on Chr11 that explained 4.97–9.31% of phenotypic variation. Of these, two QTLs for EP were expressed over 2 years. Three previously reported QTLs for protein content and seed weight were located in this region [35, 37, 38]. Seven QTLs were located in a physical position (43923975–45,138,371 bp) on Chr19, of which *qPH19a*, *qNN19a* and *qPH19b-2* have large effect (28.01, 32.56, 24.49%) on phenotypic variation in comparison to the others. Mansur et al. found two QTLs associated with protein and oil were close to this region [55]. Orf et al. also reported that this locus as associated with seed weight [40]. In addition, three QTLs (*qBN19a, qPH19b-1, qEP19b*) were detected on Chr19 (40662371–40,701,058 bp) in this study. Similar loci have been previously reported for seed weight, protein and oil content [43, 46, 56]. Another two QTLs (*qIP19a, qSW19a-2*) on Chr19 were mapped to the interval of 42,309,067–42,469,449 bp. Some of the seed weight QTLs were detected near this position in past studies [40, 45, 56, 57]. Moreover, QTLs for protein and oil content were also previously identified in this region by both Orf et al. and Qi er al. [40, 43]. Interestingly, in this study, highly significant correlations were observed among PH, NN, BN, EP and SW. QTL mapping analysis showed that these traits were all linked to same region on three chromosomes (Chr04, Chr06, Chr19), which is consistent with the conclusion of phenotypic correlation analysis, and provided a genetic explanation for these associations. These QTL clusters may be cause of the pleiotropism or associations between the traits related. Every single cluster may function as an independent gene or closely linked genes [58]. More importantly, some of those QTLs on Chr04, Chr06, Chr11, and Chr19 were identified in both years. These chromosome regions can be considered robust and environmentally stable, which could be helpful for further studies aimed at simultaneously altering soybean yield and quality in a predictable manner.

### Three candidate genes on Chr19

Based on the predicted function of the five groups, three predicted genes (*Glyma19g37910, 37,570, 36,990*) were selected as the best candidate genes that may affect both yield and quality because they are involved in various biological process (Table 4). *Glyma19g37910* encodes a member of the basic leucine zipper transcription factor family, involved in *arabidopsis* abscisic acid signalling during seed maturation and germination. GO analysis showed that this gene participated in more than ten biological process, which include seed development, lipid storage, gibberellin biosynthesis, and vegetative to reproductive phase transition of the meristem, etc. *Glyma19g37570* gene has a domain predicted to encode a serine/threonine protein kinase that could influence cells in various ways. This gene is related to the process of stem cell division, protein phosphorylation, gibberellin biosynthesis and timing of the transition from vegetative to reproductive development. *Glyma19g36990* encodes a plastidic triose phosphate isomerase, and GO analysis revealed that this gene participates in three catabolic process (glycine, tryptophan, and glycerol) and four biosynthetic process (indoleacetic acid, cysteine, and glyceraldehyde-3-phosphate, isopentenyl diphosphate). Moreover, it also plays a key role in multicellular organism reproduction and primary root development, which may have an effect on the yield and quality of crops. In general, these three candidates should be investigated in more detail in further studies to increase our understanding regarding the factors involved in the process of improving quality and productivity in soybean.

**Table 4:** The information of three candidates’ annotations

Glyma 1.1 ID	Physical position	Arabidopsis homologues	GO Biological Process	GO Molecular Function
Glyma19g37910	44,997,977–45,003,243	AT2G36270	gibberellic acid/abscisic acid mediated signaling pathway; photomorphogenesis; positive regulation of transcription, DNA-dependent; seed development; lipid storage; vegetative to reproductive phase transition of meristem; meristem structural organization; regulation of flower development.	protein binding; sequence-specific DNA binding transcription factor activity; protein dimerization activity;
Glyma19g37570	44,674,047–44,687,995	AT5G03730	regulation of post-embryonic root development; regulation of stem cell division; regulation of timing of transition from vegetative to reproductive phase; protein autophosphorylation; negative regulation of ethylene mediated signaling pathway; gibberellin biosynthetic process.	transferase activity, transferring phosphorus-containing groups; kinase activity
Glyma19g36990	44,249,954–44,254,095	AT2G21170	primary root development; glyceraldehyde-3-phosphate biosynthetic process; regulation of protein localization; multicellular organism reproduction; glycerol catabolic process; cysteine biosynthetic process; indoleacetic acid biosynthetic process; chloroplast organization.	catalytic activity; glycolysis

The three candidate genes were selected in a physical position between 43,923,975 and 45,138,371 bp on Chr19. Seven QTLs (*qPH19a, qNN19a, qEP19a, qSW19a-1, qPH19b-2, qNN19b, qIP19b*) associated with five traits were all detected within this genomic region

## Conclusions

In this study, we genotyped a recombinant inbred line (RIL) population (Zhonghuang 24 × Huaxia 3) using a restriction-site associated DNA sequencing (RAD-seq) approach. A high-density soybean genetic map with 2639 recombination bins was constructed and used to identify QTLs that were shown to influence six yield-related and two quality traits. A total of 47 QTLs for six yield-related traits and 13 QTLs for two quality traits were identified. Of these, 34 QTLs detected herein were coincident with those of previous research [18, 27, 32, 34, 35, 39–50, 56, 57, 59–64]. Eighteen QTLs were stable QTLs that were identified in 2 years. Twenty-six QTLs were shown for the first time in this research, of which 10 were novel and stable QTLs. In addition, eight QTL hotspots on four chromosomes were identified for the correlated traits. Three predicted genes were selected as candidate genes that may directly or indirectly influence both yield and quality in soybean.

## Additional files

Additional file 1: The distribution of LOD values for eight traits. Maximum LOD score of each major QTL is indicated next to the peak. Red lines indicated data was collected in 2012(yr12), blue lines indicated data was collected in 2015(yr15). Different line colors indicate data collected in different years (yr12, 2012; yr15, 2015). (PDF 7993 kb)

Additional file 2: Difference in agronomic traits between the parents and their recombinant inbred lines. ^a^ The data used was generated in summer 2012; ^b^ The data used was generated in summer 2015. (PDF 37 kb)

Additional file 3: QTLs detected in RILs population that reported by previously studies. **marked by QTL name indicates a new, stable QTL that was detected in both years; ^a^Chr indicates chromosome; ^b^LOD indicates the logarithm of odds score; ^c^ Percentage of phenotypic variation explained; ^d^ Related QTLs have been reported in the previous studies of the region which was identified in the Zhonghuang 24 and Huaxi 3 RILs population. (PDF 63 kb)

Additional file 4: 8 QTL hotspots detected in Zhonghuang24 × Huaxia3 RIL population in 2 years. **marked by QTL name indicates a new, stable QTL that was detected in both years; ^a^Chr indicates chromosome; ^b^LOD indicates the logarithm of odds score; ^c^Percentage of phenotypic variation explained. (PDF 62 kb)

Additional file 5: Annotation description of five gene groups based on GO analysis. (PDF 28 kb)

## Abbreviations

BN: Number of branches on main stem
Chr: Chromosome
CIM: Composite interval mapping
EP: Number of effective pod
GO: Gene ontology
IP: Number of invalid pod
LOD: Logarithm of the odds
NN: Number of nodes on main stem
PH: Plant height
QTL: Quantitative trait loci
SNP: Single nucleotide polymorphisms
SW: 100-seed weight
R^2^: Phenotypic variance
RAD-seq: Restriction-site associated DNA sequencing
RIL: Recombinant inbred line
