# Supplementary figures and images for: Construction of high-density genetic map and QTL mapping of yield-related and two quality traits in soybean RILs population by RAD-sequencing
Source: BMC Genomics. 2017 Jun 19;18:466. doi: 10.1186/s12864-017-3854-8 (PMC5477377; doi:10.1186/s12864-017-3854-8)

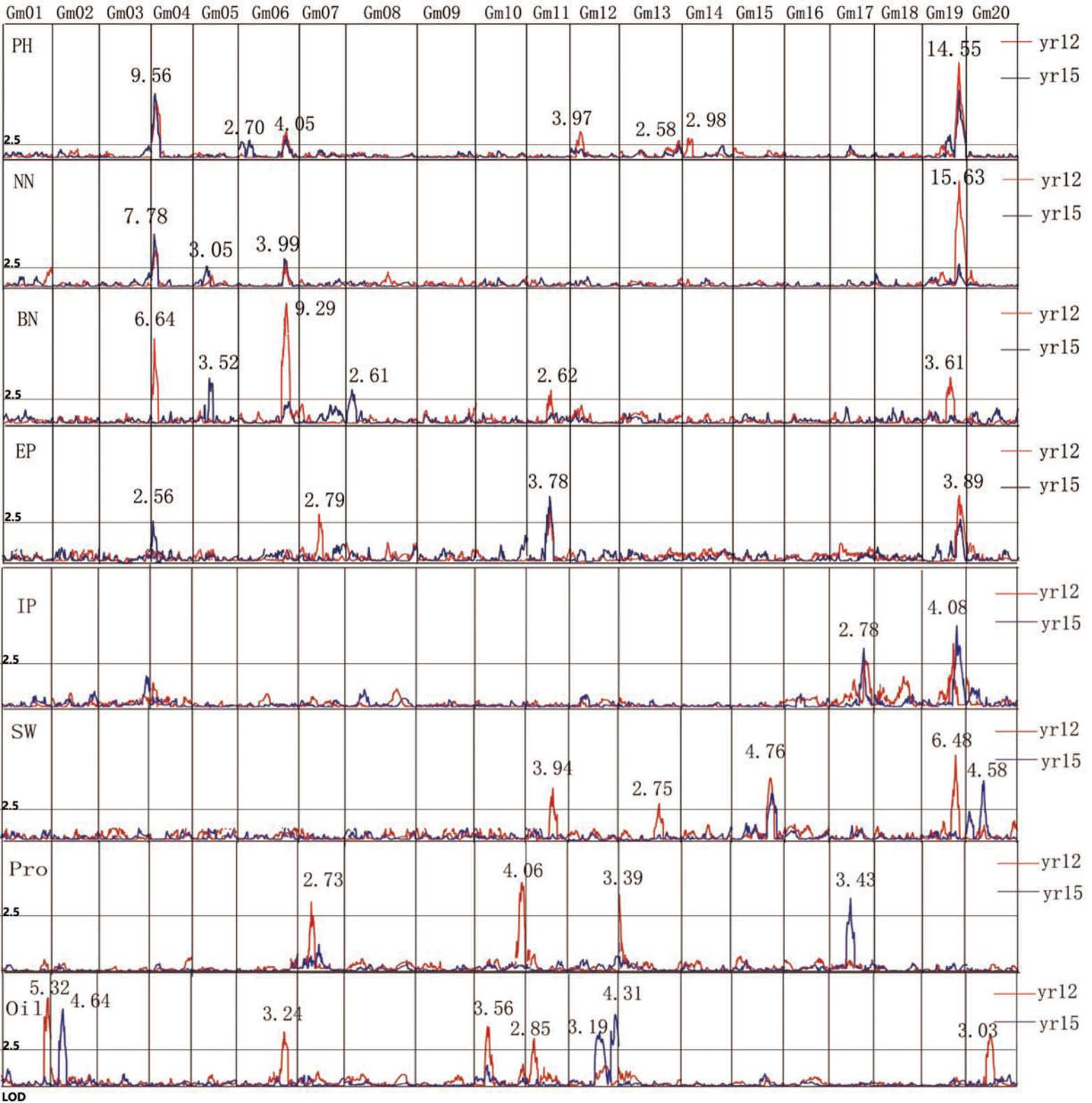

Supplement: Supplementary file 1 — The distribution of LOD values for eight traits. Maximum LOD score of each major QTL is indicated next to the peak. Red lines indicated data was collected in 2012(yr12), blue lines indicated data was collected in 2015(yr15). Different line colors indicate data collected in different years (yr12, 2012; yr15, 2015). (PDF 7993 kb) [file 12864_2017_3854_MOESM1_ESM.pdf]
