# Supplementary material for: Construction of high-density genetic map and QTL mapping of yield-related and two quality traits in soybean RILs population by RAD-sequencing
Source: BMC Genomics. 2017 Jun 19;18:466. doi: 10.1186/s12864-017-3854-8 (PMC5477377; doi:10.1186/s12864-017-3854-8)
Supplement: Supplementary file 2 — Difference in agronomic traits between the parents and their recombinant inbred lines. a The data used was generated in summer 2012; b The data used was generated in summer 2015. (PDF 37 kb) [file 12864_2017_3854_MOESM2_ESM.pdf]

Table S1. Difference in agronomic traits between the parents and their recombinant inbred lines

Traits	Parents		RIL populations		
	Zhonghuang24	Huaxia3	Means	Range	coefficient of variation/%
PH/cm(2012[a])	64.8±4.5	86.0±4.3	80.7±17.5	37.7-132.1	21.7
PH/cm(2015[b])	46.6±2.5	62.2±3.1	70.2±16.5	30.8-107.4	23.5
NN(2012[a])	15±1.1	19±1.0	17±2.5	11-24	14.7
NN(2015[b])	11±1.4	19±0.8	17±2.9	10-24	17.1
BN (2012[a])	2±0.4	8±0.4	5±1.4	1-9	28.0
BN (2015[b])	2±0.7	5±0.5	5±1.5	1-11	30.0
EP(2012[a])	47±7.3	111±14.4	73±19.8	31-223	27.1
EP(2015[b])	28±2.9	72±7.3	68±19.5	31-137	28.7
IP(2012[a])	1±0.7	8±2.1	4±3.3	1-26	82.5
IP(2015[b])	2±0.7	4±1.3	4±3.5	0-21	87.5
SW/g(2012[a])	15.0±0.2	16.9±0.2	16.3±1.9	12.1-21.5	11.7
SW/g(2015[b])	15.8±0.1	18.8±0.2	14.4±2.0	10.41-20.47	13.9
Pro (2012[a])	40.9±0.1	45.1±0.1	40.9±1.5	35.7-46.7	3.7
Pro (2015[b])	39.7±0.1	42.4±0.1	41.4±1.1	38.7-44.1	2.7
Oil (2012[a])	23.1±0.1	21.7±0.1	22.7±1.1	18.2-25.6	4.8
Oil(2015[b])	23.1±0.0	21.9±0.1	22.2±0.4	20.6-23.8	1.8

PH: plant height; NN: number of nodes; BN: number of branches; EP: number of effective pods; IP: number of invalid pods; SW: 100-seed weight; Pro: seed protein content; Oil: seed oil content

[a] The data used was generated in summer 2012

[b] The data used was generated in summer 2015
