# Supplementary material for: Construction of high-density genetic map and QTL mapping of yield-related and two quality traits in soybean RILs population by RAD-sequencing
Source: BMC Genomics. 2017 Jun 19;18:466. doi: 10.1186/s12864-017-3854-8 (PMC5477377; doi:10.1186/s12864-017-3854-8)
Supplement: Supplementary file 3 — QTLs detected in RILs population that reported by previously studies. **marked by QTL name indicates a new, stable QTL that was detected in both years; aChr indicates chromosome; bLOD indicates the logarithm of odds score; c Percentage of phenotypic variation explained; d Related QTLs have been reported in the previous studies of the region which was identified in the Zhonghuang 24 and Huaxi 3 RILs population. (PDF 63 kb) [file 12864_2017_3854_MOESM3_ESM.pdf]

Table S2. QTLs detected in RILs population that reported by previously studies

QTL Name	Chr[a]: Physical position	LOD[b]	Additive effect	R^2 (%)[c]	Related QTLs[d]
qPH06a-1	Chr06:18376759-18680015	3.34	4.64	5.57	Plant height 18-4[34]
qPH06a-2	Chr06:38809121-41420709	4.05	4.82	6.69	Plant height 20-4[35]
qPH13a	Chr13:42318489-42430858	2.58	-3.50	3.78	Plant height 23-5[44]
qPH14a-1	Chr14:2331748-2575520	2.98	4.10	4.98	Plant height 34-6[18]
qPH19a**	Chr19:44931101-45081885	14.55	-9.60	28.01	Plant height 4-2 [32]
qNN06a-1**	Chr06:18680016-18977543	2.68	0.62	5.18	Node number 2-2[64]
qNN06a-2	Chr06:38299978-38809120	3.48	0.68	6.65	Node number 1-4[35]
qBN06a	Chr06:38299978-38809120	9.29	0.64	19.77	Branching 1-4[42]
qBN19a	Chr19:40662371-40701058	3.61	0.38	7.18	Branching 3-2 [60]
qEP19a**	Chr19:44764317-44931100	3.89	6.29	9.06	Pod number 1-9[39]
qIP19a	Chr19:42309067-42469449	3.68	1.49	9.19	Pod number 1-9[39]
qSW19a-1	Chr19:43923975-44261964	6.48	-0.73	14.64	Seed weight 7-7/17-1[40,57]
qSW15a**	Chr15:14767270-16683419	4.76	0.62	10.75	Seed weight 6-1[40]
qSW11a	Chr11:18125735-18594023	3.94	-0.55	8.52	Seed weight 32-1[61]
qSW13a	Chr13:34923404-35011420	2.75	-0.45	5.74	Seed weight 45-6[62]
qSW19a-2	Chr19:42309067-42469449	3.82	-0.57	8.99	Seed weight 15-7/35-7[45,56]
qPH06b-2	Chr06:28751251-33715231	3.43	4.73	7.23	Plant height18-4[34]
qPH19b-1	Chr19:40662371-40701058	3.55	4.96	7.31	Plant height 1-1[59]
qPH19b-2**	Chr19:44931101-45081885	10.34	-9.11	24.49	Plant height 4-2 [32]
qNN06b**	Chr06:18977544-19504937	3.99	0.92	9.10	Node number 2-2[64]
qEP19b	Chr19:40662371-40701058	3.57	5.90	8.74	Pod number 1-9[39]
qIP19b**	Chr19:44261965-44544573	4.08	1.15	10.36	Pod number 1-9[39]
qSW15b**	Chr15:16683420-17383420	3.44	0.68	11.07	Seed weight 6-1[40]
qSW20b-2	Chr20:33207531-33259106	4.58	-0.77	12.80	Seed weight 9-1[63]
qPro07a	Chr07:8197411-8246204	2.73	0.39	6.83	Seed protein 24-4[45]
qOil01a	Chr01:53643709-53766235	5.32	-0.37	13.30	Seed oil 37-2[27]
qOil06a	Chr06:37764770-38299977	3.24	0.28	7.74	Seed oil 33-1[41]
qOil10a	Chr10:5306155-5360052	3.56	-0.30	8.54	Seed oil 34-6[46]

qOil11a	Chr11:5083072-5441113	2.85	-0.27	6.76	Seed oil 4-5[47]
qOil20a	Chr20:34770628-34809740	3.03	0.27	7.18	Seed oil 24-6/27-4[43,44]
qOil02b-1	Chr02:4838719-5017571	2.70	0.12	7.52	Seed oil 20-2[50]
qOil02b-2	Chr02:6519813-6826911	4.64	0.16	12.49	Seed oil 20-2[50]
qOil12b-1	Chr12:33860238-34136030	3.19	0.13	8.50	Seed oil 6-5[48]
qOil12b-2	Chr12:38572504-39497790	4.31	-0.16	12.06	Seed oil 7-1[49]

**marked by QTL name indicates a new, stable QTL that was detected in both years

[a]Chr indicates chromosome.

[b]LOD indicates the logarithm of odds score.

[c] Percentage of phenotypic variation explained.

[d]Related QTLs have been reported in the previous studies of the region which was identified in the Zhonghuang 24 and Huaxi 3 RILs population
