# Supplementary material for: Construction of high-density genetic map and QTL mapping of yield-related and two quality traits in soybean RILs population by RAD-sequencing
Source: BMC Genomics. 2017 Jun 19;18:466. doi: 10.1186/s12864-017-3854-8 (PMC5477377; doi:10.1186/s12864-017-3854-8)
Supplement: Supplementary file 4 — 8 QTL hotspots detected in Zhonghuang24 × Huaxia3 RIL population in 2 years. **marked by QTL name indicates a new, stable QTL that was detected in both years; aChr indicates chromosome; bLOD indicates the logarithm of odds score; cPercentage of phenotypic variation explained. (PDF 62 kb) [file 12864_2017_3854_MOESM4_ESM.pdf]

Table S3. 8 QTL hotspots detected in Zhonghuang24 × Huaxia3 RIL population in two years

Chr[a]_Bin range	Physical position	QTL Name	LOD[b]	Additive effect	R^2 (%)[c]	Related QTLs[d]
Chr04_bin19-bin20	3657048-3781822	qNN04a-1**	5.29	0.83	9.10	
		qBN04a	6.64	0.55	13.44	Novel region
		qNN04b**	7.78	1.33	17.68	
		qEP04b	2.56	5.18	6.17	
Chr04_bin22-bin23	3815206-5131478	qPH04a**	8.21	7.73	15.71	Seed weight 6-7[40];Seed protein 12-2[33].
		qNN04a-2	5.09	0.86	9.59	
		qPH04b**	9.56	8.78	21.53	
Chr06_bin99-bin101	18376759-19504937	qPH06a-1	3.33	4.64	5.57	Plant height 18-4[34]; Node number 2-2[64];
		qNN06a-1**	2.68	0.62	5.18	Pod number 3-3[51];Seed weight 15-1/16-1[45,52];
		qNN06b**	3.99	0.92	9.10	Seed oil 23-1[45];Seed oil plus protein 1-3[42].
Chr06_bin110-bin112	37764770-41420709	qPH06a-2	4.05	4.82	6.69	Plant height 20-4[35];Node number 1-4[35];
		qNN06a-2	3.48	0.68	6.65	Branching 1-4[42]; Seed oil 33-1/31-2 [45,53];
		qBN06a	9.29	0.64	19.77	Seed oil plus protein 1-2[42];Pod number 2-1[42];
		qOil06a	3.24	0.28	7.74	Seed weight 31-1/40-3[41,17];
						Seed protein 28-1/35-2[54,17].
Chr11_bin72-bin77	14584137-16449587	qBN11a	2.62	0.32	4.97	Seed weight 4-1/11-1[37,38];
		qEP11a**	3.07	5.52	6.96	Seed protein 25-1/25-2[35].
		qEP11b**	3.78	6.05	9.31	
Chr19_bin97-bin103	43923975-45138371	qPH19a**	14.55	-9.60	28.01	Plant height 4-2/13-8 [32,33];Pod number 1-9[39];
		qNN19a**	15.63	-1.48	32.56	Seed weight 7-7/17-1[40,57];
		qEP19a**	3.89	6.29	9.06	Seed oil 3-1/23-4[55,45];
		qSW19a-1	6.48	-0.73	14.64	Seed protein 2-2[55].
		qPH19b-2**	10.34	-9.11	24.49	
		qNN19b**	3.15	-0.76	6.60	
		qIP19b**	4.08	1.15	10.36	
Chr19_bin77	40662371-40701058	qBN19a	3.61	0.38	7.18	Plant height 1-1[59]; Pod num 1-9[39];

		qPH19b-1	3.55	4.96	7.31	Seed weight 35-7[56]; Seed protein 30-7[46];
		qEP19b	3.57	5.90	8.74	Seed oil 24-7[43].
Chr19_bin91	42309067-42469449	qIP19a	3.68	1.49	9.19	Pod num 1-9[39];Seed protein 8-1[40];
		qSW19a-2	3.82	-0.57	8.99	Seed oil 24-7[43];
						Seed weight 5-1/15-7/17-1/35-7 [40,45,57,56].

**marked by QTL name indicates a stable QTL that was detected in two years.

[a]Chr indicates chromosome.

[b]LOD indicates the logarithm of odds score.

[c] Percentage of phenotypic variation explained.

[d]Related QTLs have been reported in previous studies of the region which was identified in the Zhonghuang 24 and Huaxi 3 RILs population
