# Supplementary material for: Construction of high-density genetic map and QTL mapping of yield-related and two quality traits in soybean RILs population by RAD-sequencing
Source: BMC Genomics. 2017 Jun 19;18:466. doi: 10.1186/s12864-017-3854-8 (PMC5477377; doi:10.1186/s12864-017-3854-8)
Supplement: Supplementary file 5 — Annotation description of five gene groups based on GO analysis. (PDF 28 kb) [file 12864_2017_3854_MOESM5_ESM.pdf]

Table S4. Annotation description of five gene groups based on GO analysis

Groups	Biological Process	Glyma 1.1 ID	Annotation Description
Ⅰ	phytohormones regulation	Glyma19g36660,36761,36771,36781,36820,36990, 37000,37520,37540,37570,37910,38060,38140.	response to auxin stimulus/abscisic acid/ethylene; indoleacetic acid biosynthetic.
Ⅱ	metabolic process	Glyma19g36840,36850,36860,36910,36970,36990, 37000,37310,37410,37450,37750,37810,37820, 37830,37870,37930,37940,37031,37116.	polyamine/nuclear-transcribed mRNA/glycine/protein/fatty acid/methylglyoxal catabolic process; glycerol ether/ one-carbon/hydrogen peroxide/carbohydrate/lipid/ brassinosteroid metabolic process.
Ⅲ	protein phosphorylation	Glyma19g36900,37290,37430,37570,37770,37940.	protein polymerization.
Ⅳ	cellular process	Glyma19g36710,36720,36820,36990,37060,37250, 37480,37540,37570,37600,37610,37680,37770, 37890,38150,36791.	cell differentiation; cell proliferation; cell size; multicellular organism reproduction; cell growth; meiosis; cell division; cell cycle process.
Ⅴ	organ morphogenesis	Glyma19g36700,36710,36820,36990,37380,37530, 37570,37660,37740,38150,37910,37750,37870, 37890,37010,37410.	Regulation of plant-type hypersensitive response; seedling development; root/stamen/embryo development; leaf/flower morphogenesis; plant-type cell wall organization; regulation of meristem growth
